# The role of tumor-associated macrophages in tumor immune evasion

**DOI:** 10.1007/s00432-024-05777-4

**Published:** 2024-05-07

**Authors:** Ruizhe Huang, Ting Kang, Siyu Chen

**Affiliations:** Department of Oncology, Xin Hua Hospital Affiliated to Shanghai Jiao Tong University School of Medicine, Shanghai, 200092 China

**Keywords:** Tumor-associated macrophages, Immune evasion, Tumor microenvironment

## Abstract

**Background:**

Tumor growth is closely linked to the activities of various cells in the tumor microenvironment (TME), particularly immune cells. During tumor progression, circulating monocytes and macrophages are recruited, altering the TME and accelerating growth. These macrophages adjust their functions in response to signals from tumor and stromal cells. Tumor-associated macrophages (TAMs), similar to M2 macrophages, are key regulators in the TME.

**Methods:**

We review the origins, characteristics, and functions of TAMs within the TME. This analysis includes the mechanisms through which TAMs facilitate immune evasion and promote tumor metastasis. Additionally, we explore potential therapeutic strategies that target TAMs.

**Results:**

TAMs are instrumental in mediating tumor immune evasion and malignant behaviors. They release cytokines that inhibit effector immune cells and attract additional immunosuppressive cells to the TME. TAMs primarily target effector T cells, inducing exhaustion directly, influencing activity indirectly through cellular interactions, or suppressing through immune checkpoints. Additionally, TAMs are directly involved in tumor proliferation, angiogenesis, invasion, and metastasis.

**Summary:**

Developing innovative tumor-targeted therapies and immunotherapeutic strategies is currently a promising focus in oncology. Given the pivotal role of TAMs in immune evasion, several therapeutic approaches have been devised to target them. These include leveraging epigenetics, metabolic reprogramming, and cellular engineering to repolarize TAMs, inhibiting their recruitment and activity, and using TAMs as drug delivery vehicles. Although some of these strategies remain distant from clinical application, we believe that future therapies targeting TAMs will offer significant benefits to cancer patients.

## Introduction

### Overview of the TME

Rising evidence indicates that the TME takes on a crucial role in propelling cancer progression and dictating the response to conventional therapeutic approaches (Hiam-Galvez et al. 2021). TME is a complex and dynamic milieu consisting of tumor cells and various stromal components, all of which interact and evolve in response to each other and therapeutic interventions. A vital feature of the TME is its heterogeneity in terms of the types of cells present and their functional states.

Macrophages, specifically TAMs, constitute a significant portion of the cellular component within the TME. In numerous cancer types, including breast, ovarian, and lung cancers, a high presence of TAMs typically indicates a less favorable prognosis (Qian et al. 2009; Qian and Pollard 2010; Zhang et al. 2012). TAMs primarily arise from monocytes that migrate to the tumor location. Their differentiation is shaped by a range of elements found within the TME. After integrating into the tumor, TAMs exhibit various phenotypes similar to M2-like or activated macrophages. These M2-like TAMs are often involved in fostering tumor progression, stimulating blood vessel formation, aiding in metastasis, and hindering immune responses against the tumor. The interaction between TAMs and cancer cells plays a pivotal role in determining the tumor’s development and response to treatment. Cancer cells can emit substances that draw in macrophages and modify them to assist in tumor proliferation and invasive behavior. TAMs can secrete various growth factors, cytokines, and enzymes in return. These substances encourage tumor expansion and alter the surrounding extracellular matrix, thus aiding the invasion and spread of tumor cells.

The TME is a complex and diverse arena, hosting various interactions among cells, including macrophages like TAMs. While these cells contribute to tumor development and immune response dynamics, a comprehensive understanding of the TME’s myriad interactions is crucial. This broader perspective is vital for devising new therapeutic approaches that target the tumor and its supportive environment.

### Concept and significance of immune evasion in cancer

Tumors often employ immune evasion tactics, allowing them to slip past the host’s immune defenses, thereby increasing their chances of continued growth and survival. Mechanisms that enable the evasion of immune attack encompass the selection of tumor variants resilient to immune effectors—occasionally referred to as “immunoediting”—and the gradual establishment of an immune-suppressive milieu within the tumor (Vinay et al. 2015). It is broadly acknowledged that the immune system of the host plays a significant part in the development and progression of cancer. However, the spotlight has typically been on the capacity of immunity to eradicate tumors rather than its potential to enhance them, both of which may be vitally crucial. The complex relationship between the immune system and cancer typically involves a series of intricate events. These events ultimately lead to one of two outcomes: the successful eradication of the tumor or the tumor’s successful evasion of immune detection (Becker et al. 2013).

## Overview of TAMs

In normal conditions, macrophages are involved in starting immune responses against pathogens, maintaining tissue balance, and aiding in tissue repair and remodeling. Additionally, within the TME, they are associated with various processes like matrix remodeling, blood vessel formation, metastasis, and tumor advancement. However, their role is just one aspect of a more extensive set of interactions in the TME (Chen and Zhang 2017). Macrophages adapt their functional types following various microenvironmental signals from tumor and stromal cells. Typically, macrophages are classified into two subsets based on their activation and function: M1 macrophages, known for their traditional activation often linked to pro-inflammatory actions, and M2 macrophages, recognized for their alternative activation, contributing to anti-inflammatory activities and tissue repair and remodeling. TAMs closely resemble M2 macrophages and are pivotal in modifying the TME. Studies in clinicopathology have implied that an accumulation of TAMs within tumors is linked with unfavorable clinical outcomes. Consistent with these findings, a range of experimental and animal model studies have supported the notion that TAMs may contribute to creating a favorable environment for both the emergence and progression of tumors. This concept is reinforced by observations across different types of research, highlighting TAMs potential impact on tumor dynamics (Chanmee et al. 2014).

### Origin of TAMs

**Fig. 1:**
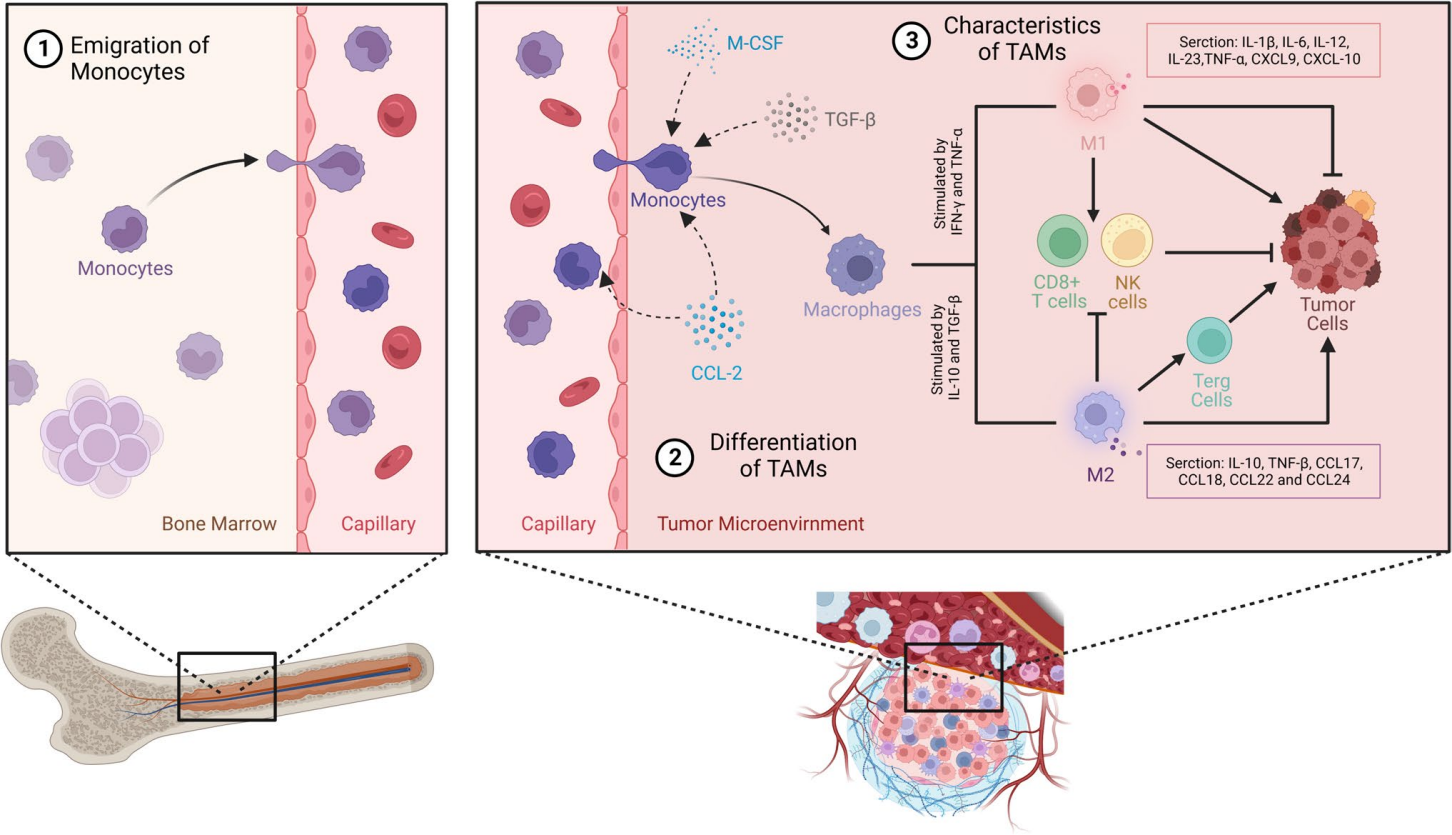
TAMs are derived from monocytes in the bone marrow. Monocytes mature in the bone marrow and then migrate to the peripheral blood circulation. Recruited by CCL-2 chemokine signals, monocytes enter the TME and differentiate into macrophages under the influence of cytokines such as M-CSF and TGF-β. The macrophages then differentiate into two phenotypes, M1 and M2, stimulated by more cytokines in the TME, and ultimately exert specific effects on tumor cells and other immune cells within the TME. The image was created using https://www.biorender.com/

Macrophages, innate immune cells found in tissues, maintain bodily equilibrium and defend against pathogens (Murray and Wynn 2011). Regarding the origin of TAMs, there are two primary sources. The first, widely recognized in academic literature and textbooks for decades, involves circulating monocytes originating from the bone marrow (Cheng et al. 2022); in the context of tumor development, monocytes are recruited to the tumor site due to the chemotactic signals emitted by tumor cells and the surrounding stroma. Once monocytes reach the TME, they transform into macrophages under the influence of various local signals. These signals include but are not limited to, macrophage colony-stimulating factor (M-CSF), transforming growth factor-β (TGF-β), and a range of chemokines. This differentiation process is influenced by the nature of cytokines present in the TME, which can lead to the development of either pro-inflammatory M1 macrophages or anti-inflammatory M2 macrophages (Amer et al. 2022) (Fig. 1).

The other category is derived from tissue-resident macrophages. In mice, macrophages can be found in numerous body regions such as the brain, skin, liver, kidney, lung, and heart. These cells primarily arise from the yolk sac or fetal liver, and once mature, their sustenance through adulthood does not rely on the presence of circulating monocyte precursors, provided there are no stress factors present. In a typical healthy liver, macrophages are primarily present as specific tissue-resident cells, termed Kupffer cells (KCs). Emerging research suggests these resident macrophages likely derive from erythromyeloid progenitors (EMPs) that express the CSF1R in either the yolk sac or fetal liver (Laviron and Boissonnas 2019). During the progression of liver cancer, these resident macrophages are activated by elements that encourage the growth of tumors. This activation triggers a change in their phenotype, eventually leading to their transformation into TAMs. Fate-mapping techniques and fluorescent markers have revealed that the composition of TAMs in different tumor models includes a diverse mix of tissue-resident and inflammatory macrophages. However, there has not been a specific functional characterization tied to their origin in varying cancer contexts (Laviron and Boissonnas 2019).

### Proportion and significance of TAMs in the TME

TAMs in the TME are often recognized as the most populous group of tumor-infiltrating immune cells, emphasizing their substantial proportion within the TME (Zhou et al. 2020a, b, c). Cancer-associated fibroblasts (CAFs), like TAMs, constitute a significant part of the cellular makeup in tumors. The interactions between CAFs and TAMs play a critical role in cancer development and the alteration of immune responses (Gunaydin 2021). TAMs attach great importance to driving tumor invasion and metastasis. They actively engage in angiogenesis, which is essential for the growth and sustenance of tumors (Goswami et al. 2017; Pan et al. 2020). Their involvement in these fundamental aspects of tumor biology underscores their significance within the TME, shedding light on how their abundance and activities contribute to the evolution of the tumoral landscape.

The immunosuppressive nature of TAMs, particularly the M2-like TAMs, is a hallmark of their significance in modulating immune responses within the TME. The preponderance of M2-like TAMs often leads to tumor immunosuppression and chemoresistance, significantly hindering the efficacy of conventional and immunotherapeutic treatment regimens (Zhou et al. 2020a, b, c). Furthermore, TAMs have been associated with drug resistance, a trait that further delineates their role in therapy evasion and underscores the challenges in cancer treatment (Pan et al. 2020). TAMs exhibit a remarkable degree of functional plasticity, manifested through their polarization into M1-like and M2-like TAMs. This functional diversity plays a critical role in dictating the immunological landscape of the TME. The ability to alter their functional phenotype also presents a potential avenue for therapeutic interventions targeting TAMs repolarization. For instance, reversing the tumor hypoxia microenvironment has aided TAMs repolarization, suggesting the prospect of developing novel therapeutic strategies targeting TAMs (Yang et al. 2020a, b). The extensive understanding of the roles and significance of TAMs within the TME has spurred the exploration of TAM-targeted cancer immunotherapies. Despite the promising strides, challenges remain in optimizing such strategies for enhanced clinical efficacy. Moreover, the expression of various molecular mediators by TAMs, including cytokines, chemokines, and growth factors, further elucidates their integral roles in tumor progression and immune modulation (Zhu et al. 2022).

The significant number and varied roles of TAMs in the TME emphasize their importance in understanding cancer behavior, immune response, and resistance to treatment. Continued research is critically important due to the high prevalence of TAMs within the TME and their complex role in promoting tumor growth and evading immune detection. This research should focus on unraveling the complex interactions between TAMs and other TME elements to leverage these insights to create new and effective cancer immunotherapies.

### Epigenetic modifications of TAMs

In oncology, epigenetic modification is one of the primary mechanisms by which the TME affects the behavior of infiltrating cells (Flavahan et al. 2017). Key epigenetic mechanisms regulating immune function include DNA methylation, RNA methylation, histone modifications chromatin accessibility, etc. Methylation, as one of the most important epigenetic modifications, explains the complex role of TAMs in the TME (Cao et al. 2023; Tien et al. 2023).

In DNA methylation, methyl groups are covalently attached to cytosines, often on CpG dinucleotides, and methylation of CpG islands in promoters often results in silencing of gene expression. In mammals, cytosine methylation is mediated by DNA methyltransferases (DNMTs), while conversion of methylcytosine to 5-hydroxymethylcytosine is by Tet methylcytosine dioxygenase (TET) catalytic (Yang et al. 2010). DNMTs tend to promote M1 polarization (Cheng et al. 2014; Yang et al. 2014), but TET proteins are associated with the M2 phenotype. Depletion of Tet2 in tumor-associated macrophages reduces immunosuppressive functions and inhibits melanoma growth in vivo (Pan et al. 2017). Therefore, the opposing effects of DNMT and TET on DNA methylation are to regulate the balance between the M1 and M2 states of macrophages (Tien et al. 2023). Analysis of tissue pathology has found that DNMT3B is overexpressed in breast and colorectal cancer (Girault et al. 2003; Nosho et al. 2009), and DNMT inhibitors (DNMTi), such as decitabine and 5-azacytidine (5-AC) can sensitize cancer cells to various therapeutic drugs (Stone et al. 2017; Travers et al. 2019).

Similarly, adenine in RNA can also be methylated to form N6-methyladenosine (m6A), which significantly impacts various biological behaviors such as RNA splicing, stability, and translation (Zhou et al. 2020a, b, c). Typical methyl additions are catalyzed by written complexes composed of multifunctional subunits. Methyltransferase-like 3 (METTL3) and methyltransferase-like 14 (METTL14) form a heterodimer as the core of the writing complex; the former has catalytic capabilities, while METTL14 allosterically activates METTL3 and promotes RNA binding (Zhou et al. 2021). m6A methylation regulates dynamic macrophage activation. METTL3-driven methylation positively regulates macrophage activation by accelerating the decay of IRKAM transcripts that inhibit TLR signaling (Tong et al. 2021). Furthermore, METTL3 promotes M1 macrophage polarization through m6A-mediated enhancement of STAT1 expression. METTL14 maintains negative feedback control of TLR4/NF-κB signaling by inducing SOCS1 attenuation, preventing macrophage overactivation (Du et al. 2020). m6A methylation promotes macrophage polarization and regulates inflammation. METTL14 deficiency induces M2 macrophage polarization. It has been shown that loss of METTL14 in TAMs leads to CD8 + T cell dysfunction. METTL14 specifically regulates C1q + TAMs, and depletion of METTL14 in C1q + TAMs promotes Ebi3 mRNA accumulation through the METTL14-YTHDF2 axis, leading to a transition of intratumoral CD8 + T cells toward a dysfunctional state (Dong et al. 2021).

## Subtypes of TAMs and their biological characteristics

In 1987, Hibbs found that macrophages might kill tumor cells through the arginine metabolism pathway (Hibbs et al. 1987). Further studies suggested that nitrous oxide, a product of arginine metabolism, was responsible for killing tumor cells (Hibbs et al. 1988). In another study, tumor growth was found to be different in different types of mice, such as BL6 germ line inhibition and BALB/C germ line tolerance (Lifsted et al. 1998). This study and subsequent results led people to think that the macrophage phenotype is different in different types of mice. Consequently, certain immunologists have introduced the Th1 and Th2 immunity concept, which hinges on arginine metabolism’s equilibrium. The Th1 type tends to generate nitric oxide, whereas the Th2 type is inclined to produce ornithine(Mills 1991, 1992). Following this bifurcation, Mills and colleagues categorized macrophages into M1 and M2 types (Mills et al. 2000). Various studies have identified specific cell surface markers, like the macrophage mannose receptor 1 (MRC1) present on M2 but absent on M1 macrophages, as indicators to differentiate between these two cell types (Murray et al. 2014). However, the classification of macrophages remains a topic of debate. Diverse factors influence their phenotypes and activation states, including signaling molecules, growth factors, transcription factors, and epigenetic and post-transcriptional changes.

Additionally, environmental cues such as cytokines, cell-to-cell interactions, and metabolites play a role in this variability (Chen and Zhang 2017; Collins et al. 2021; T’Jonck et al. 2018). Macrophages can modify their activation state. Activating macrophages is critical in maintaining tissue balance and developing and progressing inflammation and disease. Generally speaking, M1 macrophages are essential in driving inflammatory responses and fighting against tumors, whereas M2 macrophages promote anti-inflammatory effects and support tumor growth (Chanmee et al. 2014). However, the role of TAMs in different tumor tissues is complex, with TAMs characterized by different markers influencing various aspects such as cancer typing, clinical staging, prognosis, etc. Here, we summarize the phenotype and function of TAMs in some common tumor types (Table 1).

**Table 1:** Markers and functions of tams in different tumor tissues

Types of cancer	TAMs markers	TAMs Function	References
Breast cancer	CD68-Pan macrophages CD11c-M1 HLA-DRα-M1 CD163-M2 CD206-M2 MMP9-M2	CD68 + TAMs are an independent predictor of reduced overall survival (OS) and relapse-free survival (RFS) CD163 + TAMs are positively associated with poor histological grade, larger tumor size, and lymph node (LN) metastasis	(Sousa et al. 2015; Riabov et al. 2016;Koru-Sengul et al. 2016; Tiainen et al. 2015; DeNardo et al. 2011; Gordon and Pluddemann 2017)
Colorectal cancer	CD68-M1 NOS2-M1 CD163-M2 CD206-M2	CD68 + TAMs are associated with high patient survival CD163 + TAMs suggest earlier local recurrence and poorer survival NOS2 + TAMs are significantly associated with improved cancer-specific survival (CSS)	(Edin et al. 2012; Nakayama et al. 2002; Sickert et al. 2005; Cavnar et al. 2017; Shabo et al. 2009)
Non-small cell lung cancer	CD68-Pan macrophages HLA-DR-M1 iNOS-M1 pSTAT1-M1 CD163-M2 CD204-M2 MACRO-M2	CD68 + TAMs correlated with a higher TNM stage, peritumoral lymphatic vessel density (LVD), and LN metastasis CD163 + TAMs accumulation is strongly associated with reduced progression-free survival (PFS) CD204 + TAMs correlated with tumor differentiation, pathologic stage, T status, nodal involvement, lymphatic permeation, vessel invasion, and pleural invasion MACRO + TAMs contribute to an immunosuppressive mechanism protecting cancer cells	(Almatroodi et al. 2016; Rakaee et al. 2019; Zhang et al. 2011; Yusen et al. 2018; Li et al. 2018; Yang et al. 2015; La Fleur et al. 2018)
Ovarian cancer	CD68-Pan macrophages CD80-M1 CD163-M2	CD68 + TAMs are significantly associated with lower OS CD163 + TAMs are negative predictors of PFS and OS	(Lan et al. 2013; No et al. 2013; Yin et al. 2016)
Prostate cancer	CD68-Pan macrophages YKL40-M1 CD163-M2	CD68 + TAMs are associated with TNM clinical stage and are predictors of shorter CSS CD163 + TAMs are associated with a higher incidence of metastasis and a lower incidence of CSS	(Lundholm et al. 2015; Takayama et al. 2009)

### Characteristics and functions of M1 macrophages

Understanding the traits and roles of M1 macrophages within the TME is vital for grasping their impact on cancer development and therapy. M1 macrophages are renowned for their inherent abilities in phagocytosis, predation, and lysis and for the anticancer effects stemming from their intensified inflammatory response against tumors (Liu et al. 2021). M1 macrophages get triggered by toll-like receptors (TLRs) or Th1 cytokines like TNF-α, IFN-γ, and CSF2. They exhibit pro-inflammatory, microbicidal, and antitumor properties. Standard markers for identifying The markers of M1 macrophages comprise HLA-DR, CD11, CD80, CD86, nitric oxide synthase (iNOS), YLK-40, and pSTAT1(Jayasingam et al. 2019; Zhou et al. 2020a, b, c). M1 macrophages can enhance the ability of CD8 + T cells and NK cells to emit tumor cells or induce tumor cell apoptosis by secreting TNF, IL-6, IL-12, and other cytokines (Dungan et al. 2014). However, this antitumor mechanism cannot avoid the immune escape of cancer stem cells (Luen et al. 2017). Cancer stem cells often survive through the filtration of M1 macrophages (Lu et al. 2014).

### Characteristics and functions of M2 macrophages

M2 macrophages generally promote tumor growth and aid in tumor immune evasion, associated with a poor prognosis. Their markers primarily include CD163, CD204, CD206, MMP9, and MACRO (Larionova et al. 2020). It’s understood that M2 macrophages are divisible into four distinct subtypes. Th2 cytokines like IL-4 and IL-13 induce the M2a phenotype in macrophages, while the M2b type is prompted by the activation of TLRs in conjunction with immune complexes. The M2c subtype is a result of IL-10 polarization. However, within the TME, macrophages are typically represented by the M2d phenotype, better known as TAMs. (Hao et al. 2012; Sica and Mantovani 2012). In contrast to M1 macrophages, M2 macrophages have a low expression of IL-12, a high expression of IL-10, and a low antitumor activity.

#### Expression of Anti-inflammatory Molecules and Immune Regulation

M2 macrophages release molecules that have anti-inflammatory properties, like IL-10 (Wang et al. 2024). This substance reduces the production of pro-inflammatory cytokines in T cells and NK cells, thereby fostering an environment that is more conducive to the growth of tumors (Yang et al. 2023a, b). Additionally, these macrophages generate TGF-β, a compound that inhibits the functions of various immune cells and encourages the transformation of effector T cells into regulatory T cells, known as Tregs (Gao et al. 2022a, b; Xue et al. 2020). This process further diminishes the immune system’s response to tumor cells (Perez and Rius-Perez 2022). Arginase 1(Arg-1) is another molecule produced by M2 macrophages, which creates an immunosuppressive environment by metabolizing L-arginine, thereby impairing T cell function and promoting tumor growth (Viola et al. 2019). M2 macrophages inhibit the activation and proliferation of T cells and facilitate the conversion of effector T cells into Tregs, known for their immunosuppressive functions (Bhattacharya and Aggarwal 2019; Chen et al. 2023). Although the exact interaction between M2 macrophages and B cells needs further exploration, macrophages have been shown to secrete cytokines BAFF and APRIL, which are crucial for plasma cell isotype switching, indicating a form of interaction with B cells.

M2 macrophages are key players in promoting tumor-friendly conditions in cancer. They do this through several mechanisms: regulating both angiogenesis and lymph angiogenesis, suppressing immune responses, inducing hypoxic conditions, and aiding in both the proliferation and the metastasis of tumor cells (Boutilier and Elsawa 2021). Their interactions within the TME, which includes various entities like cancer cells, stromal cells, T cells, myeloid-derived suppressor cells (MDSCs), and neutrophils, are shaped by the genetic and epigenetic alterations occurring in the cancer cells (Sadhukhan and Seiwert 2023). TAMs offer vital support to cancer cells in terms of nutrients and growth factors, which contribute to the progression of the disease and the development of resistance to treatments (Vitale et al. 2019). M2 macrophages are crucial in initiating and promoting tumor growth, spread, and the ability of the tumor to evade immune detection. This role is driven by their response to cytokines, such as IL-4 and IL-13, produced by Th2 cells (Gao et al. 2022a, b).

#### Tissue repair and healing

M2 macrophages’ secretion of various growth factors and enzymes facilitates tissue repair and restores tissue architecture post-injury (Xia et al. 2023). Research by Fernandes et al. showed that the conditioned media from macrophages, derived from human umbilical cord blood, increased the osteogenic differentiation in mesenchymal stem cells (MSCs) from adipose tissue. This enhancement rely on the macrophages’ secretion of OSM (Fernandes et al. 2013). In a separate study, Gong et al. explored how mouse bone marrow macrophages influenced bone development in mouse bone marrow MSCs using an indirect transwell co-culture system. Within this setup, it was observed that M1 macrophages had reduced activity, while M2 macrophages notably enhanced the osteogenesis process mediated by MSCs (Gong et al. 2016).

Exosomes are extracellular vesicles released by cells, and their contents can play a role in intercellular communication (Kalluri and LeBleu 2020). Their essential role in health and disease has attracted much attention (Yu et al. 2022). M2 macrophage-derived exosomes also target inflammation and show anti-inflammatory properties in inflammatory diseases (Wu et al. 2020). In addition, in a previous study, MEs significantly promoted the macrophage phenotype shift from a pro-inflammatory M1 phenotype to an anti-inflammatory M2 phenotype, thereby accelerating skin wound healing (Kim et al. 2019). These properties enable M2-exosomes to play an essential role in skin wound healing induced by diabetes (Zeng et al. 2023a, b). Moreover, MEs cause the transformation of M1 macrophages into M2 macrophages by stimulating the PI3K/AKT pathway, thus significantly regulating the bone immune microenvironment thereby accelerating the healing of diabetic fractures (Wang et al. 2023).

#### Extracellular matrix remodeling

The extracellular matrix (ECM) consists of an intricate mesh of non-cellular elements that features structural proteins, with collagens being the most common, as well as matricellular proteins like periostin, thrombospondins, osteopontin, and SPARC (secreted protein acidic and rich in cysteine) (Giussani et al. 2019; Soliman et al. 2021). ECM remodeling is marked by alterations in these proteins’ content, activity, and crosslinking, affecting signal transduction. In many cancer tissues, this remodeling is signified by enhanced collagen production and accumulation, often with increased activity of enzymes like matrix metalloproteinases (MMPs), lysyl oxidase (LOX), lysyl oxidase-like proteins (LOXLs), and WNT1-inducible signaling pathway proteins (WISPs), among others (Sangaletti et al. 2017). These enzymes specifically target ECM components, catalyzing reactions that influence tissue stiffness and cell–matrix interactions through their distinct biochemical and physical characteristics (Long et al. 2022).

In collaboration with tumor cells, TAMs, especially M2 macrophages, help create a pro-carcinogenic environment. Triggered by signals from cancer cells or the ECM, TAMs play a crucial role in modifying the matrix, aiding in the directional movement of cancer cells (Liguori et al. 2011). They actively remodel the ECM through extensive matrix breakdown and production of ECM proteins. The lack of TAMs notably reduces the density and cross-linking of collagen, particularly diminishing the expression of collagen types I and XIV in cancer-associated fibroblasts (CAFs) (Afik et al. 2016). The assembly of the ECM is a crucial and highly controlled step in the process of tissue repair. When the group of ECM is impaired, it often results in fibrosis, a significant health concern that contributes to a high morbidity and mortality rate (Yoshimura 2024; Zhao et al. 2022). Fibrosis can affect many tissues, including the liver, kidney, lungs, heart, and skin. According to prevailing research, M1 macrophages are generally recognized as initiators of the healing process, whereas M2 macrophages are considered to facilitate the resolution of healing (Spiller and Koh 2017). In cases where the wound healing process is prolonged or does not correctly conclude, a pathological form of fibrosis, driven by Th2 responses and mediated by M2 macrophages, is commonly believed to occur (Wynn and Barron 2010).M2 macrophages promote tissue remodeling and angiogenesis within the TME, contributing to tumor progression ( Liu et al. 2022). They can remodel the TME through interactions with other cells, impacting their number, activity, and phenotype associated with drug resistance (Wang, et al. 2021). M2 macrophages express MARCO, which triggers a sequential remodeling of the endothelium-interstitial matrix, forming a pre-metastatic niche in the microfluidic TME (Cendrowicz et al. 2021). M2 macrophages also express enzymes such as MMP-2, MMP-7, MMP-9, MMP-11, MMP-12, and cyclooxygenase-2, which are involved in matrix remodeling and regulation of angiogenesis (Egawa et al. 2013; Hao et al. 2017; Lin et al. 2021). The secretion of MMPs from M2 macrophages, particularly the high expression of MMP-11, plays a crucial role in facilitating cancer cell metastasis, with an overexpression of MMP-11 in M2 macrophages (Saeidi et al. 2023; Zhang et al. 2016). This overexpression increases monocyte recruitment and promotes the migration of HER2 + breast cancer cells through the CCL2/CCR2/MAPK pathway, underscoring the significant impact of TAM-derived MMP-11 on the progression and metastatic potential of breast cancer (Kang et al. 2022).

### Metabolic reprogramming of TAMs

TAMs are the most abundant immune cells within TME, pivotal in immunosuppression (Christofides et al. 2022). TAMs exhibit high plasticity, capable of altering their phenotype and function in response to various environmental stimuli. Similarly, interactions with components of the TME affect the polarization of TAMs and induce metabolic reprogramming. The metabolic changes in TAMs, in turn, promote tumor progression and immune tolerance. M1 and M2 macrophages display distinct metabolic characteristics involving carbohydrate, amino acid, and lipid metabolism, significantly impacting their immune functions (O’Neill et al. 2016). The metabolic crosstalk between TAMs and the TME provides multiple targeting opportunities for therapeutic strategies (Zheng et al. 2020).

#### Glucose Metabolism Features of TAMs

One hallmark of cancer cell metabolism is the Warburg effect, whereby tumor cells exhibit inefficient glucose utilization even in the presence of ample oxygen, preferring glycolysis over oxidative phosphorylation (OXPHOS) for energy production (Koppenol et al. 2011; Pavlova et al. 2022). Within such a TME, M1 macrophages rely heavily on glycolysis to combat pathogens and tumor cells. The metabolic intermediates of aerobic glycolysis can re-enter the oxidative pentose phosphate pathway (PPP), facilitating NADPH oxidase (NOX) activity by generating nicotinamide adenine dinucleotide phosphate (NADPH), thereby producing NADPH-dependent reactive oxygen species (ROS) (Kennel and Greten 2021; Tsai et al. 2022). The relationship between M2 macrophages and glycolysis, while less prominent than M1 macrophages, is still important regarding their metabolic activity and function. Traditionally, M2 macrophages underwent metabolic reprogramming, which preferentially depended on fatty acid oxidation and mitochondrial metabolism, while glycolysis decreased (Zeng et al. 2023a, b). Intermediates produced during glycolysis may also be involved in regulating the function of M2 macrophages. For example, lactic acid produced by cancer cell glycolysis can induce the upregulation of hypoxia-inducible factor-1 alpha (HIF-1α) in TAM, thereby enhancing the expression of glycolysis gene and M2-like state, thereby promoting tumor progression, angiogenesis and epithelial-mesenchymal transformation (EMT) (Colegio et al. 2014).

In the complex environment of aerobic respiration within mitochondria, the tricarboxylic acid (TCA) cycle plays a pivotal role. However, upon M1 polarization, macrophages undergo a significant metabolic shift, displaying a truncated TCA cycle with two notable disruptions (Li and Tian 2023). Initially, the cycle is interrupted at the aconitase (ACO) step, leading to an accumulation of citrate and itaconate, known for their roles in metabolic regulation and immune response modulation. This alteration supports the cell’s switch towards a highly glycolytic phenotype, characterized by increased glycolysis and reduced mitochondrial activity, effectively adapting to the rapid energy demands of inflammatory responses (Jha et al. 2015). The second break occurs at the succinate dehydrogenase (SDH) step, further contributing to this metabolic reprogramming by promoting the stabilization and expression of HIF-1α (Tannahill et al. 2013; Vitale et al. 2019). HIF-1α is an important orchestrator in cellular responses to low oxygen levels, actively promoting the shift towards glycolytic energy production pathways (Semenza 2003). Moreover, HIF-1α plays a vital role in shaping the immunosuppressive and angiogenesis-promoting phenotype of TAMs (Corzo et al. 2010; Doedens et al. 2010). Together, these adaptations underscore the metabolic flexibility of M1 macrophages, enabling them to meet the energy demands of their role in the immune response while navigating the challenges of varied tissue microenvironments (Li and Tian 2023). These metabolic alterations lead to increased citrate and α-ketoglutarate (α-KG) levels, thereby exerting anti-inflammatory effects within the TME (Kelly and O’Neill 2015).

#### Amino Acid and Lipid Metabolism Features of TAMs

The reprogramming of amino acid metabolism is important in the phenotypic polarization of TAMs, impacting tumor development and the response to immunotherapy. TAMs limited in amino acids tend towards an anti-tumor phenotype, characterized by decreased infiltration, reduced tumor growth, and improved outcomes in immunotherapy (Penny et al. 2016). Distinct differences in amino acid utilization are observed between M1 and M2 macrophages: M1 macrophages convert L-arginine into nitric oxide via iNOS, fostering inflammation, whereas M2 macrophages use L-arginine to produce ornithine and urea, aiding in tissue repair (Chang et al. 2001; Rath et al. 2014). The tumor-promoting traits of TAMs are often linked to heightened Arg1 expression, which reduces nitric oxide levels and supports tumor growth (Arlauckas et al. 2018). This effect is evident in in vitro studies, where TAMs with increased ARG1 expression promoted the growth of breast cancer cells through elevated ARG1 activity and decreased nitric oxide production (Chang et al. 2001). Arginine is an essential amino acid for the human body and is widely involved in the immune regulation of T cells, B cells, and macrophages (Zhang et al. 2022). L-arginine is a rapid metabolite that activates T cells, which can be quickly absorbed by activated T cells, enhancing their viability and anti-tumor activity (Rodriguez et al. 2007). Arg1 inhibits the cytotoxicity of T cells by depleting L-arginine, shifting T cell metabolism towards OXPHOS, ultimately contributing to tumor progression (Geiger et al. 2016).

One of the metabolic characteristics of cancer is elevated lipid metabolism (Swierczynski et al. 2014). Increased fatty acid (FA) metabolism is shown in activated macrophages, which polarizes tissue macrophages toward the M2 phenotype. Furthermore, higher intracellular acetyl-CoA concentration may contribute to FA production in TAMs (Latour et al. 2020). CD36 is a special transporter that helps absorb exogenous FA from the environment. It breaks it down to provide a carbon source for fatty acid oxidation (FAO), fueling the TCA cycle and supporting OXPHOS, thereby generating more energy for TAMs (Huang et al. 2014). Following M2 polarization, genes involved in FA uptake, lipolysis, and FA synthesis are subsequently upregulated. FAO supports the pro-tumor potential of TAMs, as inhibiting FAO may inhibit tumorigenesis by promoting the anti-tumor properties of TAMs (Niu et al. 2017).

### The balance between M1 and M2 and its impact on tumors

The balance between M1 and M2 macrophages in the TME plays a pivotal role in the progression and treatment of cancer. M1 macrophages, known for their antitumor properties, exhibit intrinsic phagocytosis and enhanced antitumor inflammatory responses (Liu et al. 2021). On the other hand, M2 macrophages, often associated with TAMs, promote tumor growth through various mechanisms like immune suppression, angiogenesis, neovascularization, and stromal activation (Boutilier and Elsawa 2021). They contribute to pro-tumorigenic outcomes through angiogenic and lymphangiogenic regulation, immune suppression, hypoxia induction, and tumor cell proliferation.

The M1/M2 macrophage ratio within the TME significantly impacts tumor development and progression. A higher M2/M1 ratio, indicating a predominance of M2 macrophages, is often associated with poor prognosis in many solid tumors. As cancer progresses, the M1/M2 ratio of TAMs tends to decrease, and this ratio is inversely correlated with the size of the residual tumor site (Wang et al. 2021; Zhang et al. 2014). Additionally, measuring the ratio of M1/M2 macrophages could serve to assess macrophage polarization in clinical settings. This ratio offers a more biologically meaningful measure for predicting cancer outcomes than individually evaluating the quantities of M1 or M2 macrophages (Shikanai et al. 2023). Recent research underscores the importance of understanding and potentially modulating the M1/M2 balance in the TME. In particular, it has been observed that tumor-derived microparticles can induce the polarization of TAMs into an M2 state via a pathway reliant on lysosomes. Notably, encapsulated chemotherapy drugs can reverse this polarization, converting M2 macrophages back to M1 by altering lysosomal reactive oxygen species, pH levels, and calcium release. This suggests a potential new strategy for therapy (Tang et al. 2022). Another study highlighted the potential of d-lactate, a gut microbiome metabolite, in modulating M2 TAMs to an M1 phenotype, remodeling the immunosuppressive TME of hepatocellular carcinoma (Han et al. 2023). Moreover, β-element was found to regulate the M1-M2 macrophage balance through the ERK pathway (Zhou et al. 2022), underlining the various molecular pathways involved in macrophage polarization. The transformation from M2 to M1 macrophages has been demonstrated to induce a tumor-suppressive effect, highlighting the therapeutic potential of modulating macrophage polarization in cancer treatment (Duan and Luo 2021; Zhou et al. 2020a, b, c)​. This transition can lead to more favorable clinical outcomes, and specific genes are closely associated with M1 macrophages, demonstrating a potential molecular basis for macrophage-related antitumor immunity (Xu et al. 2022a, b, c).

The M1/M2 macrophage balance presents a promising avenue for cancer research and treatment, potentially serving as both a prognostic biomarker and a therapeutic target. Understanding and manipulating the M1/M2 macrophage balance within the TME might develop new cancer treatment and prognosis evaluation strategies, opening doors for more personalized and effective cancer therapies.

### Distinctions between human and murine TAMs

In murine models, TAMs exhibit distinct properties compared to their human counterparts. These differences are primarily evident in the roles of specific molecular, the process of macrophage polarization, and the characteristics of cells post-polarization. CSF significantly influences monocyte-to-macrophage differentiation, with its effects differing between mice and humans (Jeannin et al. 2018). M-CSF is ubiquitously expressed in mice, whereas granulocyte–macrophage colony-stimulating factor (GM-CSF) concentrates at inflammation sites, including tumors, produced by activated cells and tumors themselves (Hamilton et al. 2016). GM-CSF in mice drives myeloid precursors to become dendritic cells, supporting anti-tumor responses (Helft et al. 2015). It’s used in dendritic cell vaccine trials and anti-tumor therapies (Yan et al. 2017). In contrast, M-CSF and GM-CSF promote monocyte differentiation into macrophages in humans. M-CSF induces regulatory macrophages for homeostasis, and GM-CSF generates inflammatory macrophages, secreting IL-6, IL-1β, and TNF-α, with IFN-γ enhancing this production, potentially aiding tumor growth (Duluc et al. 2007; Jeannin et al. 2018; Lacey et al. 2012).

Human and mouse macrophage subsets exhibit significant phenotypic differences. Human M1 macrophages are characterized by high expression of CD80 and CD86, and secretion of inflammatory factors and IL-12, whereas mouse M1 macrophages express CD11c, CD11b, and CD38, and produce nitric oxide and pro-inflammatory cytokines (Biswas and Mantovani 2010; Raggi et al. 2017; Viola et al. 2019). In contrast, human M2 macrophages are marked by elevated expression of CD163 and CD206, producing IL-10 and growth factors, while mouse M2 macrophages express Arg-1 and VEG (Fu et al. 2020; Genin et al. 2015; Malfitano et al. 2020). The overexpressed genes in the presence of IL-4 or IFN-γ differ markedly between human and mouse macrophages, highlighting significant molecular-level differences (Ingersoll et al. 2010; Martinez et al. 2013). The typical markers for mouse M1 and M2 cells are inducible nitric oxide synthase and Arg1 expression, respectively, neither is expressed in human macrophages (Raes et al. 2005). Moreover, most genes that characterize mouse M1 and M2 cells have unknown functions, complicating the extrapolation of their roles in tumors. Supporting these findings, Zilionis et al. effectively showed that TAMs in lung tumors display distinct profiles based on their species, highlighting the critical need to study human macrophages directly rather than making assumptions based on mouse data (Zilionis et al. 2019).

## Role of TAMs in immune evasion

TAMs are a significant component of the TME, often correlating with poor prognosis and drug resistance, including resistance to immunotherapies (Kumari and Choi 2022). They exhibit a pro-tumorigenic and immunosuppressive phenotype, especially in advanced cancers, significantly influencing tumor growth, invasion, and immunosuppression (Li et al. 2022). One of the critical pathways through which TAMs mediate immune evasion is the modulation of the PD-1/PD-L1 signaling axis. This mechanism is central to developing immune checkpoint blockade therapies (Pu and Ji 2022).

Recent studies have shed light on the complex roles TAMs play in promoting immune escape. They have been found to dampen adaptive immune responses by releasing various cytokines, chemokines, and enzymes, which can directly or indirectly suppress immunity (Couper et al. 2008; Ruffell et al. 2014). This action of TAMs significantly changes the composition of immune cells within the TME, reducing the number of immune cells that fight tumors and increasing those that suppress immune responses, thereby facilitating cancer growth (Petty et al. 2021). Additionally, TAMs inhibit the activation of CD8 + T cells through several strategies, including hindering their migration to the tumor, depleting crucial nutrients needed for T cell growth, emitting anti-inflammatory cytokines, and triggering checkpoints that inhibit T cell activity.

TAMs are not a homogeneous population but exhibit phenotypic and functional diversity. Some TAMs can promote tumor progression, while others might demonstrate antitumor activities, reflecting the complexity of TAM-tumor interactions and their impact on immune evasion strategies (Pittet et al. 2022). This nuanced understanding of TAMs underscores their central role in orchestrating immune evasion and highlights the potential of targeting TAMs as a promising strategy in cancer immunotherapy (Kumari and Choi 2022). The following subsections will delve deeper into the immunosuppressive functions of TAMs, exploring how they interact with other immunosuppressive cell types and their relationship with tumor immune checkpoints to mediate immune evasion.

TAMs are pivotal contributors to the immunosuppressive milieu within the TME that facilitates cancer immune evasion. Their presence in the TME often correlates with poor prognosis, tumor progression, and a decreased efficacy of therapeutic interventions, including immunotherapies. TAMs exhibit a broad spectrum of functions that contribute to immunosuppression, primarily through the secretion of various immunosuppressive cytokines, chemokines, and enzymes. These secreted factors can directly or indirectly dampen the antitumor responses of adaptive immune cells such as T cells and NK cells (Tie et al. 2022). Additionally, TAMs interact with and promote the activities of other immunosuppressive cell populations within the TME, including Tregs and MDSCs. The synergistic actions of these cell types further augment the immunosuppressive state of the TME, providing a conducive environment for tumor growth and progression (Cheng et al. 2021; Ngambenjawong et al. 2017).

**Fig. 2:**
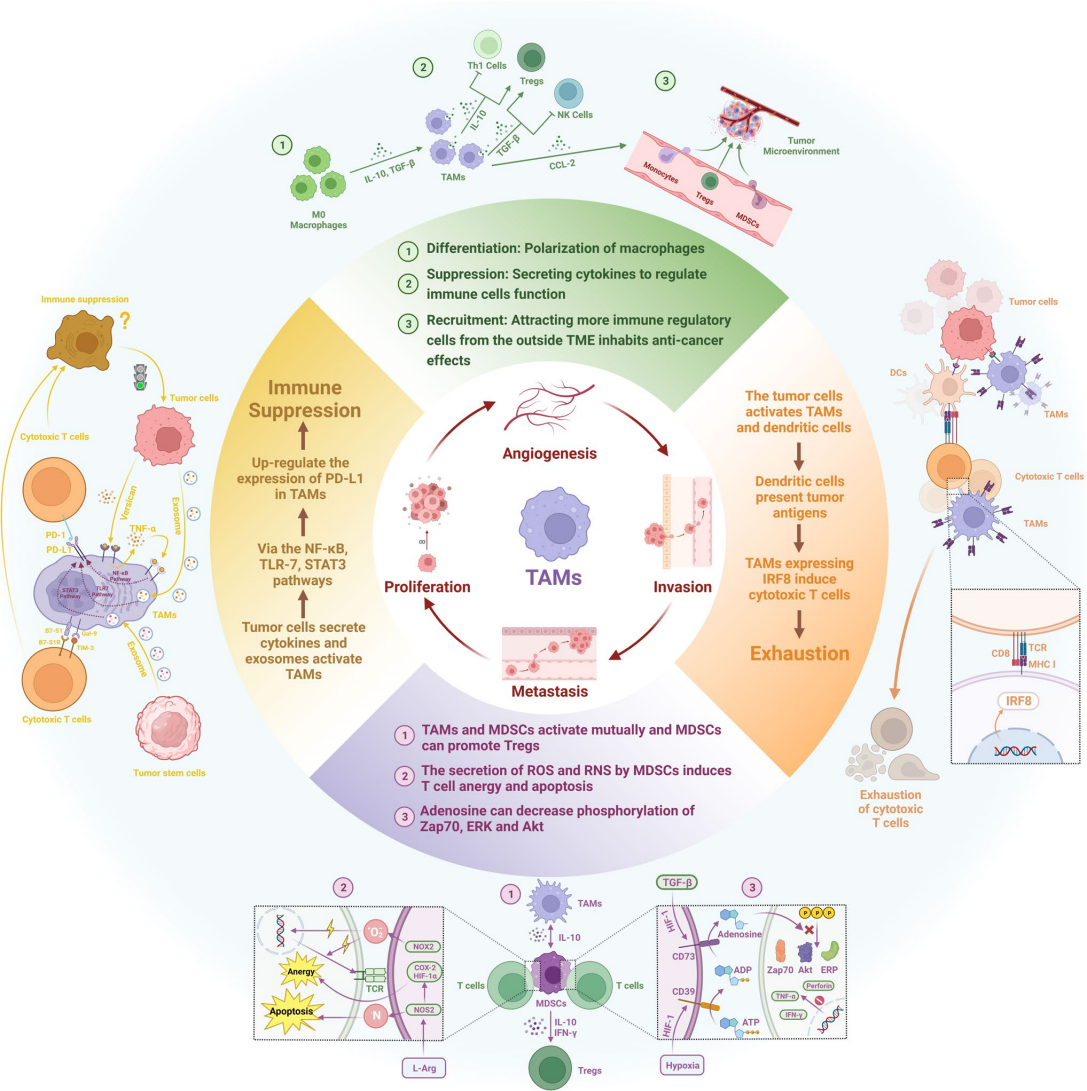
Four mechanisms of TAMs mediated tumor immune escape: **a** TAMs suppress anti-tumor immune cells and recruit immunosuppressive cells: Macrophages undergo polarization under the action of cytokines such as IL-10 and TGF-β, and modulate the function of Th1 cells, Tregs, and NK cells in the TME towards immunosuppression. TAMs can also recruit more immunosuppressive cells from the peripheral blood into the TME. **b** TAMs mediate cytotoxic T cell exhaustion: Tumor cells activate macrophages which are recognized by dendritic cells, and dendritic cells present tumor antigens to CTLs. TAMs can suppress the function of CTLs by expressing IFR8, ultimately leading to their exhaustion. **c** TAMs interact with MDSCs to induce immunosuppression: TAMs can mutually activate with MDSCs through IL-10 and activate Tergs cells. MDSCs suppress the function of T cells through various mechanisms, including the production of reactive oxygen species, reactive nitrogen species, and adenosine, ultimately leading to immunosuppression. **d** TAMs use immune checkpoints to reduce T cell function: Tumor cells and cancer stem cells activate TAMs through cytokine secretion, exosome production, and other pathways, and upregulate PD-L1 expression through signaling pathways such as NF-κB, TLR-7, and STAT3, ultimately preventing T cells from mounting an immune response against tumor cells. The image was created using https://www.biorender.com/

TAMs also play a role in modulating immune checkpoint signaling, particularly the PD-1/PD-L1 axis, thereby affecting T cell functions and the efficacy of immune checkpoint blockade therapies. The interaction between TAMs and immune checkpoint molecules contributes to T cell exhaustion and presents challenges and opportunities for therapeutic interventions to reinvigorate antitumor immune responses (Pu and Ji 2022). Moreover, the phenotypic plasticity of TAMs, which encompasses their ability to switch between pro-inflammatory (M1) and anti-inflammatory (M2) phenotypes, further complicates the dynamics of immune interactions within the TME. The protumorgenic M2 phenotype of TAMs is often predominant in tumors and is associated with immunosuppression, angiogenesis, and tissue remodeling, all favorable for tumor progression (Mantovani et al. 2017; Murray and Wynn 2011) (Fig. 2).

### Immunosuppressive factors released and their implications

TAMs secrete various immunosuppressive cytokines, prominently IL-10 and TGF-β. IL-10 functions by inhibiting Th1 cell generation and activation, reducing the production of cytokines such as IFN-γ, IL-2, and TNF-α, thereby suppressing T cell immune responses (Chen et al. 2019; Li et al. 2022). Additionally, IL-10 promotes the activation of Tregs, which further enhances immunosuppression (Xu et al. 2022a,b,c). On the other hand, TGF-β inhibits the proliferation and cytotoxic activities of T cells and NK cells while encouraging the differentiation and activation of Tregs. This mechanism facilitates tumor immunosuppression and provides a protective shield for tumor cells against immune attacks.

Besides cytokines, TAMs secrete chemokines like CCL2, which attract immunosuppressive cells such as MDSCs and Tregs to the TME, thereby augmenting tumor immunosuppression. CCL2 also facilitates tumor-associated inflammation, creating a conducive environment for tumor growth and metastasis (Zhou et al. 2020a, b, c). MMPs degrade extracellular matrix components, providing pathways for tumor cell invasion and metastasis. They also release growth factors and cytokines embedded in the extracellular matrix, further supporting tumor cell proliferation and invasion (Bied et al. 2023). Regarding immunosuppressive enzymes, Arg-1 and Indoleamine 2,3-dioxygenase (IDO) are notable. Arg-1 operates by consuming the amino acid arginine, essential for T cell metabolism and function, inhibiting T cell proliferation and weakening antitumor immune responses (Kumari and Choi 2022). Conversely, IDO functions by degrading tryptophan, producing immunosuppressive metabolites like kynurenine and hydroxytryptophan that inhibit T cell activity and promote the activation and proliferation of Tregs, thus enhancing tumor immunosuppression (Xiao et al. 2023).

### Inhibition and exhaustion of T cell functions

TAMs and T cells share a crucial interaction within the TME, particularly regarding the exhaustion and inhibition of T cell functions, which play a significant role in tumor immune evasion. This relationship is underscored by a positive feedback loop between TAMs and exhausted CD8 + T cells, which exacerbates the immunosuppressive milieu within the TME, ultimately favoring tumor progression (Lubitz and Brody 2022). TAMs employ various mechanisms to induce T cell exhaustion and inhibition. One notable mechanism is antigen presentation, where TAMs presenting cancer cell antigens drive cytotoxic T cell exhaustion. The transcription factor IRF8 has been identified as a significant player in this process, showcasing the intricate molecular orchestration underlying TAMs and T cell interactions (Nixon et al. 2022). Additionally, TAMs express immunosuppressive molecules like PD-1 and PD-L1, directly promoting cytotoxic T cell exhaustion. They also modulate the expression of several cytokines and ligands, further inhibiting T cell recruitment and function reinforcing the immunosuppressive environment​(Bied et al. 2023; Pu and Ji 2022).

Furthermore, TAMs and T cells interplay with persistent, antigen-specific synaptic contacts. Rather than activating T cells, these interactions condition them towards exhaustion. Such nuanced interactions reflect the complexity of the TME and indicate the challenges posed in reinvigorating antitumor immunity (Briana et al. 2020; Kersten et al. 2022). Strategies targeting TAMs or their interactions with T cells have shown promise on the therapeutic frontier. For instance, targeting TREM2 on TAMs or inhibiting NEK2 has been observed to reduce TAMs and alleviate T cell exhaustion, favoring the immune system’s anticancer response (Binnewies et al. 2021; Lischer & Bruns 2023). Moreover, the spatiotemporal dynamics between TAMs and T cells reveal a complex interplay in the TME. A comprehensive understanding of these dynamics is imperative to develop effective therapeutic strategies. The variability in patient responses to treatments can be attributed, at least partially, to the influence of TAMs. This underscores the importance of a more in-depth investigation into the interactions between TAMs and T cells within tumor immunology (Lubitz & Brody 2022).

The plethora of studies and findings on TAMs and T cell interactions provides a rich foundation for further investigation. It’s evident that a multifaceted approach, considering the molecular, cellular, and spatiotemporal aspects of TAMs and T cell interactions, is crucial for devising innovative therapeutic strategies to overcome tumor immune evasion and improve patient outcomes.

### Promotion of other immunosuppressive cell roles

The functional Tregs, MDSCs, and TAMs are a defining feature of the immunosuppressive milieu within the TME that significantly contributes to tumor immune evasion. Tregs and MDSCs emerge as key players in this network, with their interactions dictating the extent of immune suppression and, thereby, the progression of tumors. One research has shown that Fas ligand expression in certain tumors contributes to immunosuppression and a poorer prognosis. Immunotherapeutic approaches targeting the Fas-mediated elimination of immunosuppressive Tregs and MDSCs within tumors have been explored. For instance, a combination of IL-2 and agonistic CD40 Ab (αCD40) has elicited synergistic antitumor responses with the efficient removal of Tregs and MDSCs in murine tumor models. This elimination occurs through a Fas-dependent cell death pathway, shedding light on a potential therapeutic strategy to mitigate the immunosuppressive effects of Tregs and MDSCs within the TME (Weiss et al. 2014).

MDSCs arise from abnormal myeloid progenitor differentiation in the bone marrow, which inhibits the immune responses mediated by T cells, natural killer cells, and dendritic cells. This abnormal differentiation promotes the generation of Tregs and TAMs, driving immune escape and ultimately leading to tumor progression and metastasis (Zhao et al. 2023). The bidirectional crosstalk between MDSCs and Tregs contributes to immune evasion, limiting the success of immunotherapies, particularly those involving checkpoint inhibitors. This crosstalk can foster a supportive environment for tumor growth and progression, further highlighting the intertwined roles of these immunosuppressive cell populations within the TME (Haist et al. 2021).

TAMs and MDSCs can activate each other through IL-10. MDSCs can exhibit immunosuppressive effects in TME through a variety of pathways. Studies have shown that MDSCs can mediate Treg cell regeneration through IFN-γ and IL-10(B. Huang et al. 2006). MDSCs can also catalyze macrophages to the M2 phenotype and indirectly promote tumor growth (Beury et al. 2014). Besides, the interaction between MDSCs and T cells activated by TAMs plays a significant role. MDSCs can secrete ROS, which can cause harm to most cells. ROS can promote the expression of vascular endothelial growth factor (VEGF) receptors on the surface of MDSCs, which can encourage MDSCs to accumulate in TME (Kusmartsev et al. 2008). Evidence shows that induction of iNOS-dependent VEGF production is crucial in the accumulation of MDSCs in TME (Jayaraman et al. 2012). Activation of iNOS can produce reactive nitrogen (RNS), mainly nitric oxide (NO) (Raber et al. 2014), which can induce the expression of cyclooxygenase-2 (COX-2) and HIF-1α (Ku et al. 2016; Ostrand-Rosenberg and Sinha 2009). COX-2 can regulate PGE2 production (Park et al. 2006) and cause the upregulation of immunosuppressive markers such as IDO, IL-10, and Arg-1 in MDSCs, which has been demonstrated in vitro (Obermajer and Kalinski 2012). HIF-1α expression can stimulate VEGF production and promote tumor angiogenesis (Li et al. 2016). Moreover, reactive nitrogen can mediate the initiation of T cell apoptosis or nitration of the TCR to block T cell activation (Nagaraj et al. 2007). Promoting the conversion of ATP to adenosine is another mechanism by which TAMs indirectly inhibit T cell function through MDSCs (Li et al. 2017). CD39 catalyzes ATP to generate AMP, which CD73 catalyzes to develop adenosine (Linnemann et al. 2009). Adenosine can prevent the phosphorylation of Zap70, ERK, and Akt and reduce the expression of effector molecules on T cells such as perforin, IFN-γ, and TNF-α to maintain the priming of T cells (Hoskin et al. 2008).

Tregs and MDSCs are identified as significant components of the regulatory networks that facilitate tumor immune escape, significantly compromising the efficacy of current immunotherapies (Facciabene et al. 2012; Lv et al. 2022). Their presence and activity within the TME can hinder the ability of the immune system to effectively target and eliminate tumor cells, posing a significant challenge to the development and success of immunotherapeutic strategies (Hatziioannou et al. 2017; Wen et al. 2022).

### Relationship between TAMs and tumor immune checkpoints

TAMs can hinder the growth of CD8 + T cells by utilizing L-arginine metabolism through Arg-1, inducible iNOS, and the production of oxygen radicals (Molon et al. 2011; Movahedi et al. 2010). Additionally, TAMs contribute to T cell suppression by increasing programmed death-ligand 1 (PD-L1) levels and presenting various co-regulatory molecules on their surface (Noman et al. 2012). These molecules include PD-L1, PD-L2, the ligands for CTLA-4 (B7-1 and B7-2), T cell immunoglobulin and mucin-domain containing-3 (Tim-3), CD47, the V-domain Ig suppressor of T cell activation (VISTA), and B7-H4 (Calabrese et al. 2020; Mantovani et al. 2017; Swoboda and Sallman 2020). This array of molecules is linked to T cell exhaustion, a suppressive TME, and unfavorable outcomes in clinical settings.

TAMs are crucial in modulating the PD-1/PD-L1 immunosuppressive axis, inhibiting T cells recruitment and functional activity. This regulation is achieved by releasing cytokines, superficial immune checkpoint ligands, and exosomes, underlining the multifaceted immunosuppressive mechanisms employed by TAMs within the TME. The density of TAMs within the tumor milieu has been positively associated with PD-L1 expression on tumor cells, spotlighting TAMs as potential targets for combination therapies to enhance the responsiveness to immune checkpoint blockade (ICB) therapies, particularly in nasopharyngeal carcinoma (NPC) treatment (Deng et al. 2020). Recent studies have illustrated that T cell exhaustion and functional impairment within the TME decrease PD-L1 expression in tumor cells and macrophages. This underpins the reciprocal relationship between T cell functionality and PD-L1 expression modulated by TAMs, driving the immunosuppressive nature of the TME (Lin et al. 2019).

## Association of TAMs with tumor progression

### Proliferation

Significant research showed that MMP1, originating from TAMs, boosts the growth of HT-29 and Caco-2 cells by facilitating their cell cycle progression from the G0/G1 to the S and G2/M phases. This increased cell proliferation is further propelled by the activation of the MMP1/Protease-Activated Receptor 1 (PAR1) axis, which in turn activates the Mitogen-Activated Protein Kinase/Extracellular Signal-Regulated Kinase (MAPK/Erk) pathway. This illustrates a complex cytokine-mediated interaction between colon cancer cells and TAMs. Such interactions highlight the MMP1/PAR1/Erk1/2 pathway as a potential therapeutic target and a prognostic indicator in colorectal cancer treatments (Yu et al. 2021).

### Invasion

Through the selective targeting of tumor-promoting macrophages, the promotion of tumor cell invasion is significantly facilitated by the co-migration of TAMs and tumor cells ​(Dwyer et al. 2017). The secretion of various growth factors and the production of several proteolytic enzymes and motor-related proteins by TAMs significantly support the invasion and metastasis of tumors. Furthermore, TAMs express a range of cytokines, chemokines, growth factors, and protein hydrolases that enhance tumor cell proliferation and angiogenesis, significantly supporting tumor invasion. Additionally, TAM-derived inflammatory cytokines such as IL-23 and IL-17 have been implicated in triggering tumor-elicited inflammation, thereby further facilitating tumor invasion (Grivennikov et al. 2012).

### Metastasis

The propensity of tumor cells to metastasize is significantly affected by TAMs. Specifically, a subset of TAMs identified as F4/80 bone marrow (BM) precursors is known to gather in the bloodstream of individuals with tumors. These cells tend to position themselves predominantly at the invasive edge of the tumor, potentially aiding in metastasis (Consonni et al. 2021). TAMs contribute to the formation of an immuno-suppressive TME by generating a host of inflammatory mediators, growth factors, cytokines, and chemokines. This milieu significantly influences the metastatic behavior of tumor cells and can also induce multidrug resistance, portraying the profound impact of TAMs on tumor metastasis (Dallavalasa et al. 2021). Metabolic rewiring, such as PGE2 upregulated TAMs and glutamine metabolism in macrophages, has been demonstrated to play a pivotal role in promoting tumor metastasis, further emphasizing the multifaceted roles of TAMs in tumor progression (Wei et al. 2020).

### Angiogenesis

Angiogenesis, the creation of new blood vessels, is a hallmark of tumor progression. TAMs encode multiple gene products that promote angiogenesis, ensuring that tumors receive the essential nutrients and oxygen for growth (Yang et al. 2020a, b). The secretion of pro-angiogenic factors by TAMs significantly contributes to this process. Moreover, the angiogenic role of TAMs has implications for drug resistance; angiogenesis-induced chemoresistance arises due to the inefficient distribution of drugs within the tumor Shibutani et al. 2021). Additionally, TAMs involvement in angiogenesis is closely linked to metastasis, as angiogenesis supports tumor cell extravasation, intravasation, and colonization, which are crucial steps for metastatic progression (Fu et al. 2020). The nexus between TAMs and angiogenesis has a profound impact on tumor growth and extends to the metastatic propensity of tumors as well (Cassetta and Pollard 2023).

One of the significant mechanisms through which TAMs contribute to angiogenesis is their interaction with the innate immune system and endothelial cells. Recent research accentuates the regulatory role of this crosstalk in modulating the TME, which profoundly influences angiogenesis (Ebeling et al. 2023). The multifaceted interactions between TAMs, endothelial cells, and the innate immune system components underscore a complex regulatory network that governs angiogenesis, pivotal for tumor progression. Moreover, the abundance of TAMs within the TME showcases their significance in tumor angiogenesis. Accounting for a substantial portion of the stromal cells in the TME, TAMs epitomize an immunosuppressive M2-like phenotype in advanced cancer stages. This phenotype is instrumental in promoting tumor growth, invasion, migration, and angiogenesis, thereby offering a rationale for developing TAM-targeting therapies as part of anticancer strategies (Li et al. 2022).

Identifying new angiogenic regulators produced by TAMs holds promise in the quest for novel therapeutic targets. Once fully characterized, these regulators could potentially open new avenues for targeting tumor angiogenesis, offering hope in the battle against cancer. The recent discovery and characterization of such angiogenic regulators exemplify the rapid advancements in understanding the multifaceted roles of TAMs in tumor angiogenesis and progression (Larionova et al. 2021).

The burgeoning understanding of the intricate mechanisms through which TAMs regulate angiogenesis provides a robust foundation for developing innovative therapeutic strategies. Targeting the angiogenic regulators produced by TAMs or modulating the interactions between TAMs and other cellular constituents within the TME could potentially herald a new era of anticancer therapies. By comprehensively understanding the TAM-angiogenesis nexus, the scientific community moves closer to devising efficacious therapeutic interventions to curtail tumor progression and metastasis.

## Potential therapeutic strategies targeting TAMs

The TAMs have emerged as a promising avenue to potentiate antitumor immunity and improve cancer treatment outcomes. The heterogeneous nature of TAMs and their dynamic interactions within the TME create a complex scenario that presents challenges and opportunities for therapeutic interventions. In light of their pivotal roles in promoting tumor growth, angiogenesis, metastasis, and immunosuppression, devising strategies to modulate TAM activity or exploit their functionalities could substantially augment the efficacy of cancer therapies.

### Repolarizing TAMs phenotypes

The transition of TAMs from a pro-tumoral M2 phenotype to an anti-tumoral M1 phenotype is considered a viable therapeutic strategy (Lopez-Yrigoyen et al. 2021). Several factors, such as signaling pathways, are associated with TAMs polarization, and strategies targeting TAMs repolarization to the M1 pro-inflammatory phenotype are being discussed for cancer therapy (Gao et al. 2022a, b). Starting from different angles, methods to repolarize TAMs involve epigenetic regulation, metabolic regulation, genetic modification, inhibition of intrinsic immune checkpoints, etc.

#### TAMs repolarization by epigenetic intervention

Applying epigenetic intervention to repolarize macrophages to the M1 state may be a potential solution to the low phenotypic stability in the TME. For example, exogenous expression of some epigenetic regulators (such as DNMT1 and DNMT3B) has been reported to enhance M1 polarization of macrophages, while silencing of others (such as TET2 and PRMT2) may delay M2 polarization (Tao et al. 2023). Infusion of M1 macrophages alone can lead to increased distal metastasis of pancreatic cancer cells in mice, in which endogenous macrophages have been depleted, and M1 macrophages will be converted into TAM once they penetrate the TME while using Preconditioning of infused macrophages with DNMTi inhibits the metabolic function of TAMs and significantly reduces metastasis (Zhang et al. 2021). Systemic administration of DNMTi DAC to colon cancer mice stimulates TAM activation toward an M1-like phenotype. This is due to DAC binding to the ATP-binding cassette transporter A9 and inducing cholesterol accumulation, thereby increasing p65 phosphorylation and IL-6 expression in a DNMTi-independent manner (Shi et al. 2022). Using DNMTi epigenetic therapy to repolarize TAMs can improve the immune microenvironment from the perspective of tumor-infiltrating T cells by reactivating the expression of immune surveillance-related genes in tumor cells, and the combination with immunotherapy is more beneficial (Gonda et al. 2020; Lai et al. 2018).

#### TAMs repolarization by metabolic reprogramming

Metabolic changes are one of the drivers of macrophage suppression in the TME, so metabolic reprogramming can provide opportunities for TAM repolarization to activate tumor immunity (Mehla and Singh 2019). Glutamine synthase (GS) is a key enzyme that drives M2-like macrophage differentiation by increasing glutamine levels. Ablation of GS promotes M1-like reprogramming in TAMs and leads to CTL accumulation (Palmieri et al. 2017). Studies show that GS inhibition by methionine sulfoximine (MSO) biases M2 macrophages toward an M1-like phenotype in IL10-treated macrophages (Palmieri et al. 2017). GS inhibition induces metabolic rewiring involving glucose shunting into the TCA cycle and succinate accumulation. A low α-KG/succinate ratio enhances M1 macrophage activation. In contrast, a high ratio favors M2 macrophage function, and modulating the αKG/succinate ratio can be used to tune the immune response of TAMs (Liu et al. 2017). TAM metabolic reprogramming may also be achieved by regulating arginine catabolism. Inhibition of ARG1 by CB-1158 shifts the TME towards a pro-inflammatory environment by attenuating myeloid cell-mediated immune suppression (DeNardo and Ruffell 2019). Selective inhibitors of iNOS enhance M1 macrophage polarization; whereas NO donors inhibit M1 macrophage polarization (Lu et al. 2015).

#### Reprogramming TAMs by using CAR-M

CAR-T cell therapy has achieved significant breakthroughs in treating intractable hematologic malignancies, such as acute lymphoblastic leukemia and diffuse large B-cell lymphoma. Still, it has proven ineffective against solid tumors (Depil et al. 2020). Consequently, developing other immune cells for solid tumor treatment using the CAR platform is emerging, with the CAR-M technology proposing a new immunotherapy strategy (Lei et al. 2024). Given the abundant infiltration of macrophages in the TME, CAR-M technology can modulate the phagocytic function of macrophages, enhance their antigen presentation activity, and block them in the M1 phenotype, thus improving the immunosuppressive microenvironment (Santoni et al. 2021). Klichinsky et al. developed a robust gene transfer method, using an adenovirus vector (Ad5f35) to deliver a first-generation CAR encoding the CD3ζ signaling transduction domain to the human macrophage THP-1 cell line, thereby designing sustained pro-inflammatory signaling in macrophages within the human TME (Klichinsky et al. 2020). Recently, researchers developed second-generation M-Cars by integrating intracellular CD3ζ and TIR domains to construct antigen-targeted M-Cars, and genetically modified iMAC using second-generation M-Cars in two different solid tumor models, significantly improving the efficacy of CAR and antigen-dependent anti-tumor in vitro and in vivo (Lei et al. 2024).

### Inhibiting recruitment and activation of TAMs

Inhibiting the recruitment or proliferation of TAMs is a strategy that’s gaining traction. Various methods exist for inhibiting TAMs recruitment and inducing TAMs exhaustion, including inhibiting CSF-1R, blocking CCL2/CCR2, and targeting CD40, among others (Zhu et al. 2021). Targeting TAMs for cancer treatment includes promoting phagocytosis of TAMs to tumor cells. Although CSF-1R inhibitor PLX3397 exerts anticancer effects by inhibiting the recruitment of TAMs, signaling also regulates macrophage proliferation and activation (Li et al. 2022). Other strategies in this realm include limiting monocyte recruitment, targeting TAMs activation, reprogramming TAMs into antitumor activity, and targeting TAMs-specific markers (Pan et al. 2020). Current methods are mainly divided into two types: inhibiting pro-tumor TAMs, including inhibiting TAM recruitment and depleting TAMs, and activating antitumor TAMs, which refers to reprogramming pro-tumorigenic macrophages into anti-tumorigenic macrophages (Zhang et al. 2020). Although TAMs targeting strategies focused on macrophage depletion and inhibition of their recruitment have shown limited therapeutic efficacy, trials are still underway with combination therapies (Lopez-Yrigoyen et al. 2021).

### Utilizing TAMs as vehicles for drug delivery

The utilization of TAMs as vehicles for drug delivery is an innovative approach harnessing the inherent traits of these immune cells to enhance cancer treatment efficacy. TAMs, crucial constituents of the TME, play pivotal roles in supporting tumors and conferring therapy resistance, making them prime targets for drug delivery systems (Yang et al. 2020a, b). One promising strategy involves reprogramming TAMs by loading drugs that regulate their polarization or promote their depletion. This approach aims to initiate normalization of the TME, thereby preventing cancer progression (Yang et al. 2023a, b). The effectiveness of drug delivery can be further enhanced by employing nanoparticle-based delivery platforms, which overcome various challenges associated with conventional delivery methods (Kumar et al. 2020).

## Conclusion and outlook

TAMs are the most abundant immune cells in the TME and play a crucial role in the immunological state within the TME. TAMs have the characteristic ability to polarize into M1 and M2 subtypes, displaying almost opposite biological behaviors. The ratio of M1/M2 is an important indicator affecting tumor immunity. Thus, TAMs, along with other immune cells, constitute the complex immune microenvironment of tumor tissue. The epigenetic regulation and metabolic reprogramming of TAMs are key to explaining their functions and therapeutic targets. TAMs play a significant role in promoting tumor immune escape. Cancer cells, TAMs, and T cells form an interactive triangle. On one hand, TAMs indirectly suppress immune cells or activate immunoregulatory cells, thereby inhibiting the function of cytotoxic T cells in killing cancer cells. On the other hand, after receiving signals from tumor cells, TAMs further turn off T cells by inhibiting their immune checkpoints. TAMs also promote various biological processes in tumor development. Targeting TAMs has been a focal point of research in tumor immunotherapy. Specifically, the re-polarization of TAMs is seen as a promising approach, where from both an epigenetic and metabolic perspective, there is potential to shift TAMs towards an M1 phenotype or reverse the M2 phenotype, ultimately improving the immunosuppressive TME. In recent years, new therapies have emerged, such as the CAR-M therapy using cell engineering to modify macrophages, considered promising in replicating the success of CAR-T therapy in solid tumors.

The biological characteristics of TAMs also pose significant challenges for anticancer immunotherapy and targeted therapy in individuals. Despite macrophages being recognized for an extended period, the past fifty years have witnessed a significant deepening in our research and understanding of these cells. Nonetheless, translating this nuanced understanding of macrophage diversity into clinically actionable insights remains a formidable challenge. The advancement of enhanced intravital imaging techniques in preclinical research, coupled with cutting-edge technologies such as spatial transcriptomics, multi-color multiplex immunofluorescence, and mass spectrometry, is set to revolutionize the detailed exploration of TME at the single-cell level. Such developments promise to enrich our comprehension of TAMs, paving the way for the broader application of immunotherapy across various cancer types.

## Data Availability

The analyses and conclusions presented in this review are based upon data extracted from previously published studies, which are all publicly accessible. Each cited work within the reference section of this manuscript has been provided with a DOI number when available, ensuring traceability and ease of access for readers. For the few references that do not have DOI numbers, these publications are available through PubMed and can be located using standard literature search techniques. This approach ensures that all data supporting the findings of this review are fully accessible to those wishing to consult the original sources.
